# Epigenetic regulation of reprogramming and pluripotency: insights from histone modifications and their implications for cancer stem cell therapies

**DOI:** 10.3389/fcell.2025.1559183

**Published:** 2025-03-03

**Authors:** Woori Bae, Eun A. Ra, Myon Hee Lee

**Affiliations:** ^1^ Department of Biochemistry and Molecular Pharmacology, New York University School of Medicine, New York, NY, United States; ^2^ Institute for Cell Engineering, Johns Hopkins University School of Medicine, Baltimore, MD, United States; ^3^ Department of Neurology, Johns Hopkins University School of Medicine, Baltimore, MD, United States; ^4^ Department of Medicine, Hematology/Oncology Division, Brody School of Medicine at East Carolina University, Greenville, NC, United States

**Keywords:** pluripotent stem cells (PSCs), histone modifications, epigenetic regulations, cancer stem cells, reprogramming

## Abstract

Pluripotent stem cells (PSCs) possess the extraordinary capability to differentiate into a variety of cell types. This capability is tightly regulated by epigenetic mechanisms, particularly histone modifications. Moreover, the reprogramming of somatic or fate-committed cells into induced pluripotent stem cells (iPSCs) largely relies on these modifications, such as histone methylation and acetylation of histones. While extensive research has been conducted utilizing mouse models, the significance of histone modifications in human iPSCs is gaining increasing recognition. Recent studies underscore the importance of epigenetic regulators in both the reprogramming process and the regulation of cancer stem cells (CSCs), which are pivotal in tumor initiation and the development of treatment resistance. This review elucidates the dynamic alterations in histone modifications that impact reprogramming and emphasizes the necessity for a balance between activating and repressive marks. These epigenetic marks are influenced by enzymes such as DNA methyltransferases (DNMTs) and histone deacetylases (HDACs). Furthermore, this review explores therapeutic strategies aimed at targeting these epigenetic modifications to enhance treatment efficacy in cancer while advancing the understanding of pluripotency and reprogramming. Despite promising developments in the creation of inhibitors for histone-modifying enzymes, challenges such as selectivity and therapy resistance continue to pose significant hurdles. Therefore, future endeavors must prioritize biomarker-driven approaches and gene-editing technologies to optimize the efficacy of epigenetic therapies.

## 1 Introduction

The discovery of PSCs and their ability to differentiate into various cell types has significantly advanced regenerative medicine. PSCs, including embryonic stem cells (ESCs) and iPSCs, have tremendous therapeutic potential due to their pluripotency and self-renewal capabilities.

Maintaining pluripotency and reprogramming somatic cells into iPSCs relies on key transcription factors such as OCT4, SOX2, and NANOG, as well as critical signaling pathways, including Wnt, TGF-beta, and FGF (Marson et al., 2008; Maherali and Hochedlinger, 2009; Mossahebi-Mohammadi et al., 2020). Additionally, many studies have demonstrated that epigenetic factors play a crucial role in sustaining pluripotency and facilitating the reprogramming of somatic cells into iPSCs. Specifically, histone modifications can alter chromatin structure and influence gene expression.

Notably, PSCs and CSCs share many similarities. Therefore, understanding how histone modifications regulate PSCs could open up new avenues for therapeutic interventions in cancer.

## 2 Histone modifications in PSCs and CSCs

Histone modifications, which include methylation, acetylation, and phosphorylation, play a vital role in regulating chromatin dynamics and gene expression in PSCs (Guenther et al., 2010; Delgado-Olguin and Recillas-Targa, 2011). These modifications primarily occur on the N-terminal tails of histones H3 and H4, impacting the structural configuration of chromatin and controlling the accessibility of transcriptional machinery to DNA (Kouzarides, 2007) (Table 1). Among these modifications, histone methylation and acetylation are particularly important for regulating the pluripotency and differentiation potential of PSCs.

**TABLE 1:** The roles of epigenetic modifications in stem cells and CSCs.

Epigenetic marker	Role in stem cells	Role in CSCs	References
DNA Methylation	Controls pluripotency and differentiation by silencing lineage-specific genes	Aberrant DNA methylation leads to self-renewal, tumorigenesis, and therapy resistance in CSCs	Smith and Meissner (2013), Baylin and Jones (2016), Li and Sun (2019)
Histone Modifications	Regulates gene expression through histone acetylation, methylation, phosphorylation, etc	Alterations in histone marks control CSC plasticity, growth, and therapeutic resistance	Kouzarides (2007), Berdasco and Esteller (2010), Kumar et al. (2022)
Chromatin Remodeling	Modulates chromatin accessibility to transcription factors, regulating self-renewal and differentiation	Aberrant remodeling sustains stem-like properties, enabling CSC survival and metastasis	Wilson and Roberts (2011), Trevino et al. (2021), Chu et al. (2024)
Non-Coding RNAs (miRNAs, IncRNAs)	MicroRNAs and long non-coding RNAs regulate stem cell fate and self-renewal	Dysregulated miRNAs/lncRNAs contribute to CSC maintenance, metastasis, and drug resistance	Wang et al. (2010), Iorio and Croce (2012), Khan et al. (2019)
Polycomb Repressive Complex (PRC)	Maintains stem cell identity by silencing differentiation-associated genes	PRC components such as EZH2 are highly expressed in CSCs, promoting an undifferentiated state	Wen et al. (2017), Guo et al. (2021), Parreno et al. (2022)
Histone Demethylases (KDMs)	Histone demethylases regulate the balance between pluripotency and differentiation by removing methyl groups	Dysregulated KDMs promote stem-like features and survival in CSCs	Mosammaparast and Shi (2010), Wei et al. (2017), Wang et al. (2021)
Histone Deacetylases (HDACs)	Deacetylation of histones keeps chromatin in a condensed, inactive state, regulating gene expression	Overactive HDACs in CSCs suppress tumor suppressor genes, enhancing self-renewal and survival	McCool et al. (2007), Jiang et al. (2024)
CpG Island Methylator Phenotype (CIMP)	Methylation at CpG islands in promoter regions affects gene silencing and differentiation	CIMP in CSCs leads to the silencing of key tumor suppressors, promoting aggressive tumor phenotypes	Barzily-Rokni et al. (2011)
RNA Methylation (m6A)	Modifies mRNA stability, affecting stem cell pluripotency and lineage commitment	Dysregulation of m6A promotes CSC formation, drug resistance, and tumor growth	Zhang et al. (2017), Chen et al. (2021), Wang et al. (2023)

For instance, trimethylation at lysine four on histone H3 (H3K4me3) serves as a marker commonly found at the promoters of actively transcribed genes, such as OCT4 and SOX2. These genes are critical for maintaining pluripotency and fostering an open chromatin state that facilitates gene expression (Benayoun et al., 2014). In contrast, trimethylation at lysine 27 on histone H3 (H3K27me3), mediated by the Polycomb Repressive Complex 2 (PRC2), marks silent genes like cyclin-dependent kinase inhibitor 2A (CDKN2A) and compacts chromatin into a repressive state, which inhibits transcription (Guo et al., 2021) (Table 1).

The interaction between these two marks is essential for maintaining the “bivalent” chromatin state characteristic of PSCs, where both activating (H3K4me3) and repressive (H3K27me3) marks coexist at important developmental gene promoters. This bivalency allows PSCs to remain in a poised state, ready for rapid activation or repression in response to differentiation signals (Bernstein et al., 2006).

Histone acetylation marks, particularly H3K9ac and H3K27ac, are essential for the differentiation of stem cells into specialized cell types (Creyghton et al., 2010). These acetylation marks are linked with active transcription, allowing the chromatin structure to become more open and accessible to transcription factors (McCool et al., 2007) (Table 1).

During differentiation, histone acetyltransferases (HATs) play a crucial role by adding acetyl groups to specific lysine residues on histones. This process facilitates the activation of genes necessary for lineage commitment and functional specialization. On the other hand, HDACs remove these acetyl groups, resulting in a more compact chromatin structure that represses stem cell-associated genes.

The balance between HAT and HDAC activity is vital for directing stem cells through the differentiation process, as it determines which genes are expressed and when. This dynamic regulation of histone acetylation marks influences the transcriptional landscape, guiding stem cells to assume specific fates while preventing premature differentiation (McCool et al., 2007).

During the reprogramming of somatic cells into iPSCs, significant changes occur in histone modifications, which help reset the epigenetic landscape from a differentiated state to a pluripotent one (Liang and Zhang, 2013). Repressive marks such as H3K9me3 and H3K27me3, which are abundant in differentiated cells and indicate regions of heterochromatin, must be actively removed or modified to activate pluripotency genes (Chandra et al., 2012). For example, the removal of H3K9me3 from the NANOG promoter by the lysine demethylase 4B (KDM4B) is essential for initiating reprogramming and maintaining pluripotency (Wei et al., 2017) (Table 1). Additionally, the H3K27me3 demethylase UTX plays a crucial role during the early stages of reprogramming (Mansour et al., 2012). These enzymes work together to erase differentiation-specific epigenetic memory, thus improving both the efficiency and fidelity of the reprogramming process (Dimitrova et al., 2015).

Furthermore, histone acetylation marks play a crucial role in the reprogramming process by enhancing chromatin accessibility. Studies have demonstrated that using HDAC inhibitors, such as valproic acid (VPA), increases reprogramming efficiency (Huangfu et al., 2008; Zhai et al., 2015). These inhibitors work by preventing the removal of acetyl groups, which helps maintain an open chromatin state that is favorable for activating pluripotency-associated genes (Zhai et al., 2015; Duan et al., 2019).

For example, HDAC inhibitors enhance acetylation at the promoter regions of key genes like MYC, thereby promoting the activation of essential pluripotency pathways (Kretsovali et al., 2012) (Table 1). Additionally, the balance of histone modifications is dynamically regulated by histone-modifying enzymes, which are closely controlled during the reprogramming process (Huang et al., 2015; Yang et al., 2022; Kelly et al., 2024).

One specific example is the histone methyltransferase Set1/COMPASS complex, which is responsible for the trimethylation of H3K4. This complex is upregulated during the establishment of pluripotency, facilitating the activation of genes essential for maintaining the pluripotent state (Sze et al., 2017).

CSCs are small populations of tumor cells with the unique ability to self-renew, differentiate, and drive tumor development (Batlle and Clevers, 2017). These cells are believed to contribute significantly to tumor heterogeneity, resistance to therapies, and metastasis, making them critical targets for cancer treatment (Yu et al., 2012; Rich, 2016). Similar to PSCs, the stemness potential of CSCs is heavily influenced by epigenetic modifications, particularly histone modifications, which play a key role in regulating gene expression programs necessary for maintaining their stem-like properties.

In CSCs, specific histone modifications are crucial for promoting tumor aggressiveness by preserving a gene expression profile that enhances cell survival, proliferation, and resistance to programmed cell death. These epigenetic changes enable CSCs to maintain their tumor-initiating capacity and contribute to their resistance to conventional cancer treatments (French and Pauklin, 2021; Keyvani-Ghamsari et al., 2021; Zhou et al., 2021; Chehelgerdi et al., 2023) (Table 1).

Several histone marks play a crucial role in regulating the identity of CSCs. One significant mark is H3K27me3, a repressive modification added by EZH2, which is a component of the PRC2 (Margueron and Reinberg, 2011). This mark is often overexpressed in CSCs (Wen et al., 2017; Parreno et al., 2022) (Table 1). The H3K27me3 modification silences tumor suppressor genes, such as CDKN2A, as well as differentiation-related genes, like bone morphogenetic protein 2 (BMP2). This silencing helps maintain the cells in a more stem-like, undifferentiated state (Gosselet et al., 2007; Shi et al., 2022).

In breast cancer, elevated levels of EZH2 correlate with an increased population of CSCs and a poorer prognosis, highlighting its role in promoting tumorigenesis and metastasis (Wen et al., 2017; Verma et al., 2022) (Table 1). Similarly, H3K9me3, which is catalyzed by the histone-lysine N-methyltransferase SUV39H1 (also known as KMT1A), has been associated with the repression of differentiation pathways in glioblastoma CSCs. This repression supports their self-renewal and tumor-initiating capabilities (Saha and Muntean, 2021; Li et al., 2024).

Conversely, the activation of specific histone marks, such as H3K4me3 and H3K27ac, plays a significant role in regulating CSCs. These marks are associated with the expression of genes that provide CSCs with stemness and survival advantages. For instance, H3K4me3 is enriched at the promoters of genes crucial for stem cell maintenance and cell cycle regulation, including NANOG and OCT4, in various types of cancer, such as leukemia and colorectal cancer (Deb et al., 2014; Liu et al., 2023). Additionally, acetylation of histone H3 at lysine 27 (H3K27ac) by HATs promotes an open chromatin structure at oncogene enhancers, which contributes to the aggressive characteristics of CSCs in tumors like pancreatic and ovarian cancers (Li et al., 2021; Parreno et al., 2022; Yang et al., 2022) (Table 1). The dynamic regulation of these histone modifications enables CSCs to respond to environmental cues, including stress from chemotherapy and radiation (Li et al., 2023).

## 3 Epigenetic barriers to reprogramming

Despite significant advances in reprogramming technologies, achieving high efficiency in converting somatic cells to iPSCs remains a challenge due to various epigenetic barriers. Histone modifications, which are often irregularly distributed in differentiated cells, can create a chromatin environment that resists reprogramming (Papp and Plath, 2013; Chehelgerdi et al., 2023; Costa et al., 2023). For instance, repressive histone marks such as H3K9me3 at LINE-1 retrotransposons and H3K27me3 at the promoters of key pluripotency genes, including OCT4 and SOX2, lead to a tightly packed chromatin structure that is inaccessible to the transcription factors required to initiate reprogramming (Sun et al., 2021). The persistence of these repressive marks hinders the activation of pluripotency-associated genes, ultimately reducing both the efficiency and fidelity of the reprogramming process.

Furthermore, DNA methylation at CpG islands and the presence of histone variants, such as macroH2A, contribute to the maintenance of a differentiated state, making reprogramming more challenging. For example, DNA methylation at the GATA4 promoter can inhibit its expression, which is crucial for initiating mesendoderm differentiation during reprogramming (Barzily-Rokni et al., 2011; Hatziapostolou and Iliopoulos, 2011) (Table 1). While it is important to remove or modify these repressive marks, this process is often incomplete because the activity of the involved enzymes depends on the context and cellular environment. Enzymes such as histone demethylases (like KDM4A and KDM4B, which target H3K9me3) and HATs must be precisely directed to specific genomic regions to effectively alter chromatin states (Pack et al., 2016; Young and Dere, 2021). However, this precise targeting is frequently ineffective due to the existing chromatin structure, which is influenced by the cell’s previous transcriptional history and current epigenetic landscape.

Moreover, to successfully reprogram cells into a pluripotent state, significant changes in the cell’s gene activity, or transcriptome, are required. This process involves two key steps: removing repressive marks that silence genes and adding active marks, such as H3K4me3 and H3K27ac, at the segments that control pluripotency genes (Papp and Plath, 2011; 2013). For instance, restoring H3K4me3 to the SOX2 enhancer is critical for achieving complete reprogramming (Koche et al., 2011).

However, this process is complicated by the interactions between different histone modifications. One type of modification can influence the presence or absence of another, resulting in a complex and resilient network of epigenetic changes. To address these challenges, researchers employ various strategies. These include HDAC inhibitors to enhance chromatin accessibility, DNA methyltransferase inhibitors to reduce DNA methylation, and chromatin remodelers to physically alter chromatin structure (Li and Sun, 2019) (Table 1).

Nonetheless, determining the optimal combination of these approaches can be challenging, as the epigenetic landscape varies significantly from 1 cell type to another. These variations can lead to unintended consequences, such as genomic instability or incomplete reprogramming, ultimately resulting in a mix of different cell types. This limitation can restrict the potential applications of iPSCs in medical treatments. The roles of epigenetic mechanisms in stem cells and CSCs are summarized in Table 1.

## 4 Targeting histone modifications in CSCs for therapy

**FIGURE 1:**
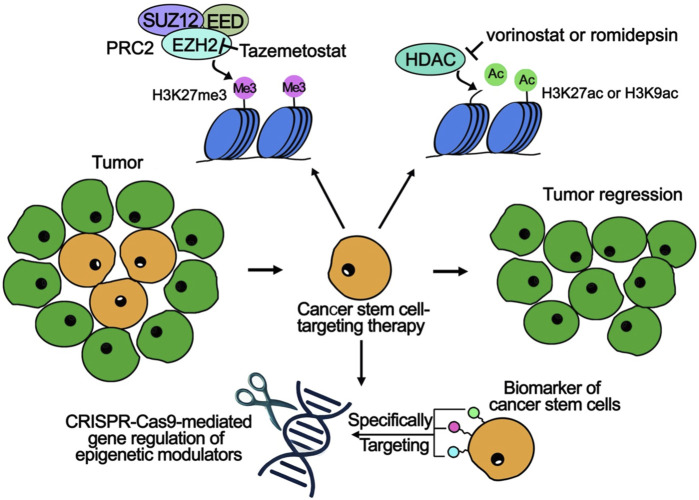
Epigenetic Modulation and Targeting Strategies for CSCs in Cancer Therapies. The upper part demonstrates two key epigenetic modifications: (1) Tazemetostat, an EZH2 inhibitor, targets the PRC2 complex (comprising SUZ12, EED, and EZH2) to reduce H3K27me3 levels and increase gene expression. (2) Vorinostat and Romidepsin are HDAC inhibitors that upregulate H3K27ac or H3K9ac levels, promoting chromatin accessibility and active transcription. The lower section highlights CSC-targeting strategies: (1) CSC biomarkers are identified and targeted to achieve selective elimination of CSC populations. (2) Epigenetic modulators are regulated using CRISPR-Cas9 to precisely modify CSC-specific pathways. The central route shows how CSC-specific therapies cause tumor regression by targeting the CSC population (orange cells), while sparing normal tumor cells (green cells).

Histone modifications play a crucial role in maintaining the characteristics of CSCs and promoting tumor progression. As a result, disrupting these epigenetic markers has become a promising strategy for cancer treatment. Recent advancements have led to the development of novel small-molecule inhibitors that specifically target key histone-modifying enzymes, including histone methyltransferases and HDACs (Kumar et al., 2023) (Table 1). These inhibitors work by dismantling the epigenetic frameworks that underpin CSC maintenance, reducing stem-like properties, promoting differentiation, and enhancing sensitivity to traditional therapies such as chemotherapy and radiation (Figure 1).

One notable advancement in cancer treatment is the development of EZH2 inhibitors, with tazemetostat being a key example that has received FDA approval for patients with both hematologic and solid tumors (Straining and Eighmy, 2022) (Figure 1). EZH2 is a component of the PRC2, which is responsible for the H3K27me3 (Figure 1). This modification is linked to gene silencing and inhibits the differentiation of mesenchymal stem cells and potential CSCs (Momparler and Côté, 2015; Straining and Eighmy, 2022) (Figure 1). High levels of EZH2 activity can repress genes associated with cell cycle arrest, promoting self-renewal in stem or progenitor cells (Kim and Roberts, 2016).

In cancer therapy, treating doxorubicin-resistant high-grade complex karyotype soft tissue sarcoma (STS) cell lines with tazemetostat has shown a reduction in the STS-CSC population. Furthermore, when tazemetostat is combined with doxorubicin, it has been found to restore chemosensitivity (O'Donnell et al., 2024). Promising results from early-phase clinical trials in cancers such as epithelioid sarcoma and follicular lymphoma highlight the potential of EZH2 inhibitors in targeting CSC populations through epigenetic reprogramming (Italiano et al., 2018).

In parallel, HDAC inhibitors like vorinostat and romidepsin have garnered attention for their ability to enhance histone acetylation, particularly at positions H3K27ac and H3K9ac, which are associated with active gene transcription (Gallinari et al., 2007) (Figure 1). By inhibiting HDAC, these compounds create a more accessible chromatin structure, allowing for the expression of genes that promote differentiation, such as p21 (CDKN1A) and BAX (Johnstone, 2002). Moreover, HDAC inhibitors increase the sensitivity of breast CSCs to treatments like cisplatin and doxorubicin across various breast cancer subtypes (Hii et al., 2020).

Vorinostat is the first FDA-approved HDAC inhibitor, specifically approved for the treatment of refractory cutaneous T Cell lymphoma (CTCL). It has been shown to reduce the expression of CSC markers and promote differentiation in glioma stem cell-like populations (GSCs) (Duvic et al., 2007; Booth et al., 2014). Additionally, Sirtuin 1 (SIRT1), the first identified member of the class III HDACs, requires NAD^+^ to catalyze the deacetylation of both histone and non-histone proteins (Liu et al., 2009). The SIRT1 inhibitor Tenovin-6 (TV-6) has demonstrated the ability to disrupt the dependence of lung adenocarcinoma CSCs on mitochondrial oxidative phosphorylation (mtOXPHOS), thereby enhancing and prolonging the therapeutic effectiveness of tyrosine kinase inhibitors (TKIs) like gefitinib (Sun et al., 2020).

Research into the potential of combining HDAC inhibitors with other therapies to overcome resistance mechanisms is ongoing. Such combinations have shown promise in increasing CSC sensitivity to radiation and chemotherapy.

Recent advances in gene therapy and single-cell epigenomic techniques are enhancing epigenetic therapies by providing detailed insights into CSC heterogeneity. Single-cell analysis allows for precise targeting of epigenetic vulnerabilities, while CRISPR-Cas9 technology is being employed to modify key epigenetic regulators involved in CSC-driven tumor growth (Xing and Meng, 2020) (Figure 1). A recent study emphasizes that the overexpression of Achaete-scute homolog 1 (ASCL1), ASCL2, and Transcription Factor AP-4 (TFAP4) significantly contributes to the regulation of CSC-like cell populations, influencing their differentiation potential based on the cellular environment through epigenetic mechanisms (Chen et al., 2023). Furthermore, haploinsufficiency of DNA methyltransferase 1 (Dnmt1) has been shown to effectively impair the self-renewal capabilities of leukemia stem cells while largely leaving normal hematopoiesis unaffected (Trowbridge et al., 2012). In the future, targeting epigenetic regulators specifically in CSCs using CRISPR-Cas9 presents a promising strategy for cancer therapies, as manipulating key factors like ASCL1, TFAP4, and Dnmt1 could disrupt CSC plasticity and differentiation, thus reducing tumorigenicity and improving treatment outcomes (Figure 1).

Despite these advancements, there are several challenges to the development of epigenetic therapies. A primary concern is the lack of selectivity—many histone-modifying enzymes, such as EZH2, are crucial not only for regulating CSCs but also for normal stem cell function. For example, studies have demonstrated that loss of EZH2 function in hematopoietic stem cells increases the likelihood of mice developing various hematologic malignancies (Mochizuki-Kashio et al., 2015). Additionally, CSCs exhibit epigenetic plasticity, allowing them to evade therapeutic interventions by activating compensatory pathways or upregulating alternative histone-modifying enzymes (Cabrera et al., 2015). This adaptability poses a significant barrier to long-term treatment success, often resulting in therapy resistance.

To address these challenges, biomarker-driven patient stratification is emerging as a promising approach that enables more personalized methods for epigenetic therapies. By identifying specific CSC markers such as CD44, CD133, ALDH, and EpCAM, clinicians can categorize patients based on the epigenetic profiles of their tumors, allowing them to select individuals who are more likely to benefit from targeted treatments (Chu et al., 2024) (Table 1). An optimal future strategy could involve the use of specific antibodies recognizing these CSC markers in combination with epigenetic-targeting agents such as tazemetostat (an EZH2 inhibitor) or vorinostat (HDAC inhibitor). This combinatorial approach may enhance therapeutic precision by selectively targeting CSC populations while minimizing off-target effects.

Furthermore, an effective strategy may involve knocking out epigenetic regulators essential for CSC self-renewal and proliferation. Advances in single-cell technologies, such as single-cell RNA sequencing and single-cell ATAC-seq, offer a valuable solution by enabling the identification of CSC-specific epigenetic signatures. Integrating this information with CRISPR-based gene editing—where Cas9 expression is regulated by CSC-specific promoters like CD133 and EpCAM—could enhance precision in modulating CSC-associated regulators while preserving normal cellular function. This strategy may contribute to the development of highly selective and efficient epigenetic therapies tailored to CSCs and their regulatory mechanisms.

Additionally, combination therapies are showing significant potential. Pairing HDAC inhibitors with other agents that target multiple epigenetic pathways has demonstrated synergistic effects in preclinical models. This combination effectively inhibits CSC functions, such as self-renewal and resistance to apoptosis (Kumar et al., 2022) (Table 1).

The next-generation of epigenetic inhibitors aims to enhance selectivity, minimize off-target effects, and improve the durability of therapeutic responses. Furthermore, gene-editing technologies like CRISPR-Cas9 are being investigated to precisely target epigenetic regulators, offering a more permanent solution for disrupting CSC plasticity.

## 5 Concluding remarks

Epigenetic therapies targeting CSCs hold significant potential for overcoming tumor growth and resistance to treatment. Advanced technologies such as single-cell epigenomic analysis and CRISPR-Cas9 gene editing allow for precise targeting of critical epigenetic regulators that support CSC adaptability and survival. Despite this progress, challenges still remain, including the non-specificity of current epigenetic drugs and the ability of CSCs to adapt and resist therapy. Utilizing biomarker-based patient stratification combined with treatment strategies may enhance therapeutic precision and minimize off-target effects. Moving forward, advancing selective epigenetic inhibitors and integrating gene-editing tools could offer more effective approaches to eliminate CSCs and improve clinical outcomes.

## References

[B1] Barzily-Rokni M. Friedman N. Ron-Bigger S. Isaac S. Michlin D. Eden A. (2011). Synergism between DNA methylation and macroH2A1 occupancy in epigenetic silencing of the tumor suppressor gene p16(CDKN2A). Nucleic Acids Res. 39, 1326–1335. 10.1093/nar/gkq994 21030442 PMC3045621

[B2] Batlle E. Clevers H. (2017). Cancer stem cells revisited. Nat. Med. 23, 1124–1134. 10.1038/nm.4409 28985214

[B3] Baylin S. B. Jones P. A. (2016). Epigenetic determinants of cancer. Cold Spring Harb. Perspect. Biol. 8, a019505. 10.1101/cshperspect.a019505 27194046 PMC5008069

[B4] Benayoun B. A. Pollina E. A. Ucar D. Mahmoudi S. Karra K. Wong E. D. (2014). H3K4me3 breadth is linked to cell identity and transcriptional consistency. Cell 158, 673–688. 10.1016/j.cell.2014.06.027 25083876 PMC4137894

[B5] Berdasco M. Esteller M. (2010). Aberrant epigenetic landscape in cancer: how cellular identity goes awry. Dev. Cell 19, 698–711. 10.1016/j.devcel.2010.10.005 21074720

[B6] Bernstein B. E. Mikkelsen T. S. Xie X. Kamal M. Huebert D. J. Cuff J. (2006). A bivalent chromatin structure marks key developmental genes in embryonic stem cells. Cell 125, 315–326. 10.1016/j.cell.2006.02.041 16630819

[B7] Booth L. Roberts J. L. Conley A. Cruickshanks N. Ridder T. Grant S. (2014). HDAC inhibitors enhance the lethality of low dose salinomycin in parental and stem-like GBM cells. Cancer Biol. Ther. 15, 305–316. 10.4161/cbt.27309 24351423 PMC3974832

[B8] Cabrera M. C. Hollingsworth R. E. Hurt E. M. (2015). Cancer stem cell plasticity and tumor hierarchy. World J. Stem Cells 7, 27–36. 10.4252/wjsc.v7.i1.27 25621103 PMC4300934

[B9] Chandra T. Kirschner K. Thuret J. Y. Pope B. D. Ryba T. Newman S. (2012). Independence of repressive histone marks and chromatin compaction during senescent heterochromatic layer formation. Mol. Cell 47, 203–214. 10.1016/j.molcel.2012.06.010 22795131 PMC3701408

[B10] Chehelgerdi M. Behdarvand Dehkordi F. Kabiri H. Salehian-Dehkordi H. Abdolvand M. Salmanizadeh S. (2023). Exploring the promising potential of induced pluripotent stem cells in cancer research and therapy. Mol. Cancer 22, 189. 10.1186/s12943-023-01873-0 38017433 PMC10683363

[B11] Chen C. Guo Y. Wu X. Si C. Xu Y. Kang Q. (2021). m6A modification in non-coding RNA: the role in cancer drug resistance. Front. Oncol. 11, 746789. 10.3389/fonc.2021.746789 34745970 PMC8564146

[B12] Chen C. C. Tran W. Song K. Sugimoto T. Obusan M. B. Wang L. (2023). Temporal evolution reveals bifurcated lineages in aggressive neuroendocrine small cell prostate cancer trans-differentiation. Cancer Cell 41, 2066–2082.e9. 10.1016/j.ccell.2023.10.009 37995683 PMC10878415

[B13] Chu X. Tian W. Ning J. Xiao G. Zhou Y. Wang Z. (2024). Cancer stem cells: advances in knowledge and implications for cancer therapy. Signal Transduct. Target Ther. 9, 170. 10.1038/s41392-024-01851-y 38965243 PMC11224386

[B14] Costa P. Sales S. L. A. Pinheiro D. P. Pontes L. Q. Maranhao S. S. Pessoa C. D. O. (2023). Epigenetic reprogramming in cancer: from diagnosis to treatment. Front. Cell Dev. Biol. 11, 1116805. 10.3389/fcell.2023.1116805 36866275 PMC9974167

[B15] Creyghton M. P. Cheng A. W. Welstead G. G. Kooistra T. Carey B. W. Steine E. J. (2010). Histone H3K27ac separates active from poised enhancers and predicts developmental state. Proc. Natl. Acad. Sci. U. S. A. 107, 21931–21936. 10.1073/pnas.1016071107 21106759 PMC3003124

[B16] Deb M. Kar S. Sengupta D. Shilpi A. Parbin S. Rath S. K. (2014). Chromatin dynamics: H3K4 methylation and H3 variant replacement during development and in cancer. Cell Mol. Life Sci. 71, 3439–3463. 10.1007/s00018-014-1605-4 24676717 PMC11113154

[B17] Delgado-Olguin P. Recillas-Targa F. (2011). Chromatin structure of pluripotent stem cells and induced pluripotent stem cells. Brief. Funct. Genomics 10, 37–49. 10.1093/bfgp/elq038 21325400 PMC3080763

[B18] Dimitrova E. Turberfield A. H. Klose R. J. (2015). Histone demethylases in chromatin biology and beyond. EMBO Rep. 16, 1620–1639. 10.15252/embr.201541113 26564907 PMC4687429

[B19] Duan Q. Li S. Wen X. Sunnassee G. Chen J. Tan S. (2019). Valproic acid enhances reprogramming efficiency and neuronal differentiation on small molecules staged-induction neural stem cells: suggested role of mTOR signaling. Front. Neurosci. 13, 867. 10.3389/fnins.2019.00867 31551670 PMC6737087

[B20] Duvic M. Talpur R. Ni X. Zhang C. Hazarika P. Kelly C. (2007). Phase 2 trial of oral vorinostat (suberoylanilide hydroxamic acid, SAHA) for refractory cutaneous T-cell lymphoma (CTCL). Blood 109, 31–39. 10.1182/blood-2006-06-025999 16960145 PMC1785068

[B21] French R. Pauklin S. (2021). Epigenetic regulation of cancer stem cell formation and maintenance. Int. J. Cancer 148, 2884–2897. 10.1002/ijc.33398 33197277 PMC8246550

[B22] Gallinari P. Di Marco S. Jones P. Pallaoro M. Steinkühler C. (2007). HDACs, histone deacetylation and gene transcription: from molecular biology to cancer therapeutics. Cell Res. 17, 195–211. 10.1038/sj.cr.7310149 17325692

[B23] Gosselet F. P. Magnaldo T. Culerrier R. M. Sarasin A. Ehrhart J. C. (2007). BMP2 and BMP6 control p57(Kip2) expression and cell growth arrest/terminal differentiation in normal primary human epidermal keratinocytes. Cell Signal 19, 731–739. 10.1016/j.cellsig.2006.09.006 17112701

[B24] Guenther M. G. Frampton G. M. Soldner F. Hockemeyer D. Mitalipova M. Jaenisch R. (2010). Chromatin structure and gene expression programs of human embryonic and induced pluripotent stem cells. Cell Stem Cell 7, 249–257. 10.1016/j.stem.2010.06.015 20682450 PMC3010384

[B25] Guo Y. Zhao S. Wang G. G. (2021). Polycomb gene silencing mechanisms: PRC2 chromatin targeting, H3K27me3 'readout', and phase separation-based compaction. Trends Genet. 37, 547–565. 10.1016/j.tig.2020.12.006 33494958 PMC8119337

[B26] Hatziapostolou M. Iliopoulos D. (2011). Epigenetic aberrations during oncogenesis. Cell Mol. Life Sci. 68, 1681–1702. 10.1007/s00018-010-0624-z 21249513 PMC11114845

[B27] Hii L. W. Chung F. F. Soo J. S. Tan B. S. Mai C. W. Leong C. O. (2020). Histone deacetylase (HDAC) inhibitors and doxorubicin combinations target both breast cancer stem cells and non-stem breast cancer cells simultaneously. Breast Cancer Res. Treat. 179, 615–629. 10.1007/s10549-019-05504-5 31784862

[B28] Huang K. Zhang X. Shi J. Yao M. Lin J. Li J. (2015). Dynamically reorganized chromatin is the key for the reprogramming of somatic cells to pluripotent cells. Sci. Rep. 5, 17691. 10.1038/srep17691 26639176 PMC4671053

[B29] Huangfu D. Maehr R. Guo W. Eijkelenboom A. Snitow M. Chen A. E. (2008). Induction of pluripotent stem cells by defined factors is greatly improved by small-molecule compounds. Nat. Biotechnol. 26, 795–797. 10.1038/nbt1418 18568017 PMC6334647

[B30] Iorio M. V. Croce C. M. (2012). MicroRNA dysregulation in cancer: diagnostics, monitoring and therapeutics. A comprehensive review. EMBO Mol. Med. 4, 143–159. 10.1002/emmm.201100209 22351564 PMC3376845

[B31] Italiano A. Soria J. C. Toulmonde M. Michot J. M. Lucchesi C. Varga A. (2018). Tazemetostat, an EZH2 inhibitor, in relapsed or refractory B-cell non-Hodgkin lymphoma and advanced solid tumours: a first-in-human, open-label, phase 1 study. Lancet Oncol. 19, 649–659. 10.1016/S1470-2045(18)30145-1 29650362

[B32] Jiang L. Huang L. Jiang W. (2024). H3K27me3-mediated epigenetic regulation in pluripotency maintenance and lineage differentiation. Cell Insight 3, 100180. 10.1016/j.cellin.2024.100180 39072246 PMC11278802

[B33] Johnstone R. W. (2002). Histone-deacetylase inhibitors: novel drugs for the treatment of cancer. Nat. Rev. Drug Discov. 1, 287–299. 10.1038/nrd772 12120280

[B34] Kelly R. D. W. Stengel K. R. Chandru A. Johnson L. C. Hiebert S. W. Cowley S. M. (2024). Histone deacetylases maintain expression of the pluripotent gene network via recruitment of RNA polymerase II to coding and noncoding loci. Genome Res. 34, 34–46. 10.1101/gr.278050.123 38290976 PMC10903948

[B35] Keyvani-Ghamsari S. Khorsandi K. Rasul A. Zaman M. K. (2021). Current understanding of epigenetics mechanism as a novel target in reducing cancer stem cells resistance. Clin. Epigenetics 13, 120. 10.1186/s13148-021-01107-4 34051847 PMC8164819

[B36] Khan A. Q. Ahmed E. I. Elareer N. R. Junejo K. Steinhoff M. Uddin S. (2019). Role of miRNA-regulated cancer stem cells in the pathogenesis of human malignancies. Cells 8, 840. 10.3390/cells8080840 31530793 PMC6721829

[B37] Kim K. H. Roberts C. W. (2016). Targeting EZH2 in cancer. Nat. Med. 22, 128–134. 10.1038/nm.4036 26845405 PMC4918227

[B38] Koche R. P. Smith Z. D. Adli M. Gu H. Ku M. Gnirke A. (2011). Reprogramming factor expression initiates widespread targeted chromatin remodeling. Cell Stem Cell 8, 96–105. 10.1016/j.stem.2010.12.001 21211784 PMC3220622

[B39] Kouzarides T. (2007). Chromatin modifications and their function. Cell 128, 693–705. 10.1016/j.cell.2007.02.005 17320507

[B40] Kretsovali A. Hadjimichael C. Charmpilas N. (2012). Histone deacetylase inhibitors in cell pluripotency, differentiation, and reprogramming. Stem Cells Int. 2012, 184154. 10.1155/2012/184154 22550500 PMC3328162

[B41] Kumar A. Emdad L. Fisher P. B. Das S. K. (2023). Targeting epigenetic regulation for cancer therapy using small molecule inhibitors. Adv. Cancer Res. 158, 73–161. 10.1016/bs.acr.2023.01.001 36990539

[B42] Kumar V. E. Nambiar R. De Souza C. Nguyen A. Chien J. Lam K. S. (2022). Targeting epigenetic modifiers of tumor plasticity and cancer stem cell behavior. Cells 11, 1403. 10.3390/cells11091403 35563709 PMC9102449

[B43] Li C. Xie Q. Ghosh S. Cao B. Du Y. Vo G. V. (2024). SUV39H1 preserves cancer stem cell chromatin state and properties in glioblastoma. bioRxiv, 2024.08.15.607856. 10.1101/2024.08.15.607856 PMC11949068 40059829

[B44] Li W. Sun Z. (2019). Mechanism of action for HDAC inhibitors-insights from omics approaches. Int. J. Mol. Sci. 20, 1616. 10.3390/ijms20071616 30939743 PMC6480157

[B45] Li W. Wu H. Sui S. Wang Q. Xu S. Pang D. (2021). Targeting histone modifications in breast cancer: a precise weapon on the way. Front. Cell Dev. Biol. 9, 736935. 10.3389/fcell.2021.736935 34595180 PMC8476812

[B46] Li Y. R. Fang Y. Lyu Z. Zhu Y. Yang L. (2023). Exploring the dynamic interplay between cancer stem cells and the tumor microenvironment: implications for novel therapeutic strategies. J. Transl. Med. 21, 686. 10.1186/s12967-023-04575-9 37784157 PMC10546755

[B47] Liang G. Zhang Y. (2013). Embryonic stem cell and induced pluripotent stem cell: an epigenetic perspective. Cell Res. 23, 49–69. 10.1038/cr.2012.175 23247625 PMC3541668

[B48] Liu R. Zhao E. Yu H. Yuan C. Abbas M. N. Cui H. (2023). Methylation across the central dogma in health and diseases: new therapeutic strategies. Signal Transduct. Target Ther. 8, 310. 10.1038/s41392-023-01528-y 37620312 PMC10449936

[B49] Liu T. Liu P. Y. Marshall G. M. (2009). The critical role of the class III histone deacetylase SIRT1 in cancer. Cancer Res. 69, 1702–1705. 10.1158/0008-5472.CAN-08-3365 19244112

[B50] Maherali N. Hochedlinger K. (2009). Tgfbeta signal inhibition cooperates in the induction of iPSCs and replaces Sox2 and cMyc. Curr. Biol. 19, 1718–1723. 10.1016/j.cub.2009.08.025 19765992 PMC3538372

[B51] Mansour A. A. Gafni O. Weinberger L. Zviran A. Ayyash M. Rais Y. (2012). The H3K27 demethylase Utx regulates somatic and germ cell epigenetic reprogramming. Nature 488, 409–413. 10.1038/nature11272 22801502

[B52] Margueron R. Reinberg D. (2011). The Polycomb complex PRC2 and its mark in life. Nature 469, 343–349. 10.1038/nature09784 21248841 PMC3760771

[B53] Marson A. Foreman R. Chevalier B. Bilodeau S. Kahn M. Young R. A. (2008). Wnt signaling promotes reprogramming of somatic cells to pluripotency. Cell Stem Cell 3, 132–135. 10.1016/j.stem.2008.06.019 18682236 PMC3235673

[B54] Mccool K. W. Xu X. Singer D. B. Murdoch F. E. Fritsch M. K. (2007). The role of histone acetylation in regulating early gene expression patterns during early embryonic stem cell differentiation. J. Biol. Chem. 282, 6696–6706. 10.1074/jbc.M609519200 17204470

[B55] Mochizuki-Kashio M. Aoyama K. Sashida G. Oshima M. Tomioka T. Muto T. (2015). Ezh2 loss in hematopoietic stem cells predisposes mice to develop heterogeneous malignancies in an Ezh1-dependent manner. Blood 126, 1172–1183. 10.1182/blood-2015-03-634428 26219303

[B56] Momparler R. L. Côté S. (2015). Targeting of cancer stem cells by inhibitors of DNA and histone methylation. Expert Opin. Investig. Drugs 24, 1031–1043. 10.1517/13543784.2015.1051220 26004134

[B57] Mosammaparast N. Shi Y. (2010). Reversal of histone methylation: biochemical and molecular mechanisms of histone demethylases. Annu. Rev. Biochem. 79, 155–179. 10.1146/annurev.biochem.78.070907.103946 20373914

[B58] Mossahebi-Mohammadi M. Quan M. Zhang J. S. Li X. (2020). FGF signaling pathway: a key regulator of stem cell pluripotency. Front. Cell Dev. Biol. 8, 79. 10.3389/fcell.2020.00079 32133359 PMC7040165

[B59] O'donnell E. Muñoz M. Davis R. Bergonio J. Randall R. L. Tepper C. (2025). Genetic and epigenetic characterization of sarcoma stem cells across subtypes identifies EZH2 as a therapeutic target. NPJ Precis. Oncol. 9 (1), 7. 10.1038/s41698-024-00776-7 PMC11717953 39789291

[B60] Pack L. R. Yamamoto K. R. Fujimori D. G. (2016). Opposing chromatin signals direct and regulate the activity of lysine demethylase 4C (KDM4C). J. Biol. Chem. 291, 6060–6070. 10.1074/jbc.M115.696864 26747609 PMC4813556

[B61] Papp B. Plath K. (2011). Reprogramming to pluripotency: stepwise resetting of the epigenetic landscape. Cell Res. 21, 486–501. 10.1038/cr.2011.28 21321600 PMC3193418

[B62] Papp B. Plath K. (2013). Epigenetics of reprogramming to induced pluripotency. Cell 152, 1324–1343. 10.1016/j.cell.2013.02.043 23498940 PMC3602907

[B63] Parreno V. Martinez A. M. Cavalli G. (2022). Mechanisms of Polycomb group protein function in cancer. Cell Res. 32, 231–253. 10.1038/s41422-021-00606-6 35046519 PMC8888700

[B64] Rich J. N. (2016). Cancer stem cells: understanding tumor hierarchy and heterogeneity. Med. Baltim. 95, S2–S7. 10.1097/MD.0000000000004764 PMC5599210 27611934

[B66] Saha N. Muntean A. G. (2021). Insight into the multi-faceted role of the SUV family of H3K9 methyltransferases in carcinogenesis and cancer progression. Biochim. Biophys. Acta Rev. Cancer 1875, 188498. 10.1016/j.bbcan.2020.188498 33373647 PMC7856268

[B67] Shi W. K. Li Y. H. Bai X. S. Lin G. L. (2022). The cell cycle-associated protein CDKN2A may promotes colorectal cancer cell metastasis by inducing epithelial-mesenchymal transition. Front. Oncol. 12, 834235. 10.3389/fonc.2022.834235 35311137 PMC8929760

[B68] Smith Z. D. Meissner A. (2013). DNA methylation: roles in mammalian development. Nat. Rev. Genet. 14, 204–220. 10.1038/nrg3354 23400093

[B69] Straining R. Eighmy W. (2022). Tazemetostat: EZH2 inhibitor. J. Adv. Pract. Oncol. 13, 158–163. 10.6004/jadpro.2022.13.2.7 35369397 PMC8955562

[B70] Sun J. Li G. Liu Y. Ma M. Song K. Li H. (2020). Targeting histone deacetylase SIRT1 selectively eradicates EGFR TKI-resistant cancer stem cells via regulation of mitochondrial oxidative phosphorylation in lung adenocarcinoma. Neoplasia 22, 33–46. 10.1016/j.neo.2019.10.006 31765940 PMC6881627

[B71] Sun L. Fu X. Ma G. Hutchins A. P. (2021). Chromatin and epigenetic rearrangements in embryonic stem cell fate transitions. Front. Cell Dev. Biol. 9, 637309. 10.3389/fcell.2021.637309 33681220 PMC7930395

[B72] Sze C. C. Cao K. Collings C. K. Marshall S. A. Rendleman E. J. Ozark P. A. (2017). Histone H3K4 methylation-dependent and -independent functions of Set1A/COMPASS in embryonic stem cell self-renewal and differentiation. Genes Dev. 31, 1732–1737. 10.1101/gad.303768.117 28939616 PMC5666672

[B73] Trevino A. E. Muller F. Andersen J. Sundaram L. Kathiria A. Shcherbina A. (2021). Chromatin and gene-regulatory dynamics of the developing human cerebral cortex at single-cell resolution. Cell 184, 5053–5069.e23. 10.1016/j.cell.2021.07.039 34390642

[B74] Trowbridge J. J. Sinha A. U. Zhu N. Li M. Armstrong S. A. Orkin S. H. (2012). Haploinsufficiency of Dnmt1 impairs leukemia stem cell function through derepression of bivalent chromatin domains. Genes Dev. 26, 344–349. 10.1101/gad.184341.111 22345515 PMC3289882

[B75] Verma A. Singh A. Singh M. P. Nengroo M. A. Saini K. K. Satrusal S. R. (2022). EZH2-H3K27me3 mediated KRT14 upregulation promotes TNBC peritoneal metastasis. Nat. Commun. 13, 7344. 10.1038/s41467-022-35059-x 36446780 PMC9708848

[B76] Wang C. Danli M. Yu H. Zhuo Z. Ye Z. (2023). N6-methyladenosine (m6A) as a regulator of carcinogenesis and drug resistance by targeting epithelial-mesenchymal transition and cancer stem cells. Heliyon 9, e14001. 10.1016/j.heliyon.2023.e14001 36915498 PMC10006539

[B77] Wang Z. Cai H. Zhao E. Cui H. (2021). The diverse roles of histone demethylase KDM4B in normal and cancer development and progression. Front. Cell Dev. Biol. 9, 790129. 10.3389/fcell.2021.790129 35186950 PMC8849108

[B78] Wang Z. Li Y. Ahmad A. Azmi A. S. Kong D. Banerjee S. (2010). Targeting miRNAs involved in cancer stem cell and EMT regulation: an emerging concept in overcoming drug resistance. Drug Resist Updat 13, 109–118. 10.1016/j.drup.2010.07.001 20692200 PMC2956795

[B79] Wei J. Antony J. Meng F. Maclean P. Rhind R. Laible G. (2017). KDM4B-mediated reduction of H3K9me3 and H3K36me3 levels improves somatic cell reprogramming into pluripotency. Sci. Rep. 7, 7514. 10.1038/s41598-017-06569-2 28790329 PMC5548918

[B80] Wen Y. Cai J. Hou Y. Huang Z. Wang Z. (2017). Role of EZH2 in cancer stem cells: from biological insight to a therapeutic target. Oncotarget 8, 37974–37990. 10.18632/oncotarget.16467 28415635 PMC5514966

[B81] Wilson B. G. Roberts C. W. (2011). SWI/SNF nucleosome remodellers and cancer. Nat. Rev. Cancer 11, 481–492. 10.1038/nrc3068 21654818

[B82] Xing H. Meng L. H. (2020). CRISPR-cas9: a powerful tool towards precision medicine in cancer treatment. Acta Pharmacol. Sin. 41, 583–587. 10.1038/s41401-019-0322-9 31792341 PMC7468325

[B83] Yang Y. Zhang M. Wang Y. (2022). The roles of histone modifications in tumorigenesis and associated inhibitors in cancer therapy. J. Natl. Cancer Cent. 2, 277–290. 10.1016/j.jncc.2022.09.002 39036551 PMC11256729

[B84] Young N. L. Dere R. (2021). Mechanistic insights into KDM4A driven genomic instability. Biochem. Soc. Trans. 49, 93–105. 10.1042/BST20191219 33492339 PMC7925003

[B85] Yu Z. Pestell T. G. Lisanti M. P. Pestell R. G. (2012). Cancer stem cells. Int. J. Biochem. Cell Biol. 44, 2144–2151. 10.1016/j.biocel.2012.08.022 22981632 PMC3496019

[B86] Zhai Y. Chen X. Yu D. Li T. Cui J. Wang G. (2015). Histone deacetylase inhibitor valproic acid promotes the induction of pluripotency in mouse fibroblasts by suppressing reprogramming-induced senescence stress. Exp. Cell Res. 337, 61–67. 10.1016/j.yexcr.2015.06.003 26112217

[B87] Zhang C. Chen Y. Sun B. Wang L. Yang Y. Ma D. (2017). m(6)A modulates haematopoietic stem and progenitor cell specification. Nature 549, 273–276. 10.1038/nature23883 28869969

[B88] Zhou H. M. Zhang J. G. Zhang X. Li Q. (2021). Targeting cancer stem cells for reversing therapy resistance: mechanism, signaling, and prospective agents. Signal Transduct. Target Ther. 6, 62. 10.1038/s41392-020-00430-1 33589595 PMC7884707

